# Fibular free flap with proximal perforator skin paddle due to aberrant anatomy — a case report

**DOI:** 10.1186/s40902-024-00416-x

**Published:** 2024-02-20

**Authors:** Kyu-Bum Kim, Jihye Ryu, Jae-Yeol Lee

**Affiliations:** Department of Oral & Maxillofacial Surgery, School of Dentistry, Pusan National University, Yangsan, Korea

**Keywords:** Proximal perforator, Fibula free flap, Vascular abnormality

## Abstract

**Background:**

The fibular free flap is considered one of the most valuable options for mandible reconstruction. A perforator flap has gained widespread acceptance in oral and maxillofacial reconstruction. Typically, the fibula flap is obtained primarily with the distal perforator due to its reliable blood supply, with less attention given to the proximal perforators during the harvesting process. Normally, the distal perforator of the fibula exhibits stability and shows limited anatomical variations. However, there have been reported cases in which the distal perforator is absent. At times, these vascular abnormalities remain undetectable through Doppler examination or preoperative angiography evaluation. Therefore, this case details the experience of encountering the rare event of vascular abnormality in oral cancer surgery.

**Case presentation:**

This article reports the case of a patient who presented with a congenital absence of the distal perforator in the peroneal artery, attributed to a vascular abnormality. Additionally, we provide a review of the concept of utilizing the proximal perforator as an alternative approach in the flap harvesting process.

**Conclusions:**

While the distal perforator of the peroneal artery is typically utilized for fibula free flap procedures, surgeons must remain cognizant of the potential for its absence due to aberrant anatomy. Recognizing an alternative approach in such cases can be pivotal for precise surgical planning and favorable outcomes in oral and maxillofacial reconstruction

## Background

In oral and maxillofacial reconstruction, the fibular free flap is one of the most useful methods [1]. In particular, the fibula free flap provides sufficient bone for reconstruction of long mandibular defects, and large diameter of the peroneal artery is advantageous for anastomosis with the neck vessels during surgery [2, 3]. Due to these advantages, it has recently been preferred flap in the reconstruction of oral and maxillofacial defects.

Recently, due to recent advances in microsurgical techniques and the widespread clinical use of perforator flaps in flap harvest, an understanding of perforator flap in reconstruction has become important in reconstructive surgery. In particular, perforator, which travels from the bone to skin paddle, is responsible for the blood supply of the flap. And to ensure the blood supply of the flap, it is important to preserve the perforator during surgical procedure.

Blood flow to the fibular free flap is supplied by perforators originating from the peroneal artery, which can be classified into proximal and distal perforator according to their distribution.

Generally, when planning reconstruction using a fibula free flap, reconstruction of the defect is planned using a perforator flap based on the distal third of the fibula, which provides excellent blood supply. However, a few cases have been reported in which conventional perforator cannot be used in the distal third due to anatomic vascular abnormalities, and clinicians should be aware of such occurrence. In this case, the patient visited the Department of Oral and Maxillofacial Surgery of our hospital and was diagnosed with squamous cell carcinoma of the right mandible. Although no evidence of abnormalities was found in preoperative Doppler examination and lower extremity angiography, we encountered vascular abnormalities with distal perforator absent of fibula in flap harvest procedure.

We report the case in which successful reconstruction was performed after harvesting a free flap using proximal perforator as alternative approach method based on anatomical understanding of perforator.

## Case presentation

**Fig. 1:**
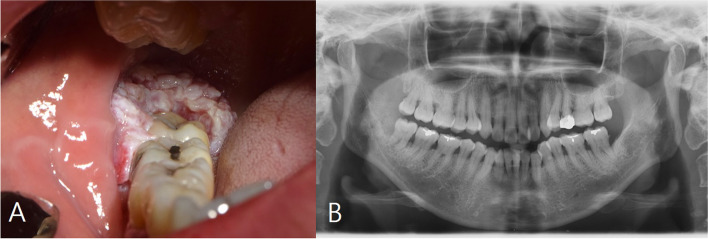
Intraoral assessment of patients in initial visit. **A** Preoperative intraoral examination presenting whitish exophytic lump on right retromolar area. **B** Preoperative panoramic radiograph view

**Fig. 2:**
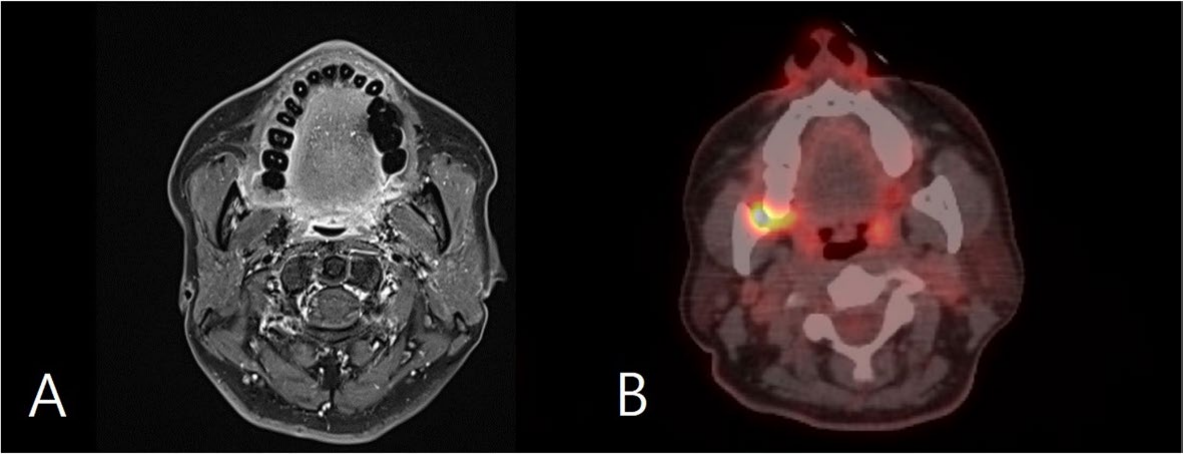
MRI and PET-CT showed irregular lesion of the right retromolar trigone region

A 55-year-old female patient visited our hospital with complaints of discomfort caused by chewing on the lower right gum for 5 months (Fig. 1) (preoperative panoramic radiograph view). Intraoral examination revealed swelling and white soft tissue formation in right retromolar trigone area. As a result of the histopathological examination, the patient was diagnosed with squamous cell carcinoma (SCC) in the lower right third molar area. Further investigation was performed to confirm the size of the lesion, and an MRI scan revealed a 1.7 × 1.4 cm soft tissue lesion in the right retromolar trigone area. On PET-CT examination, abnormally increased FDG (fluorodeoxyglucose) uptake was confirmed in the right mandible (Fig. 2).

**Fig. 3:**
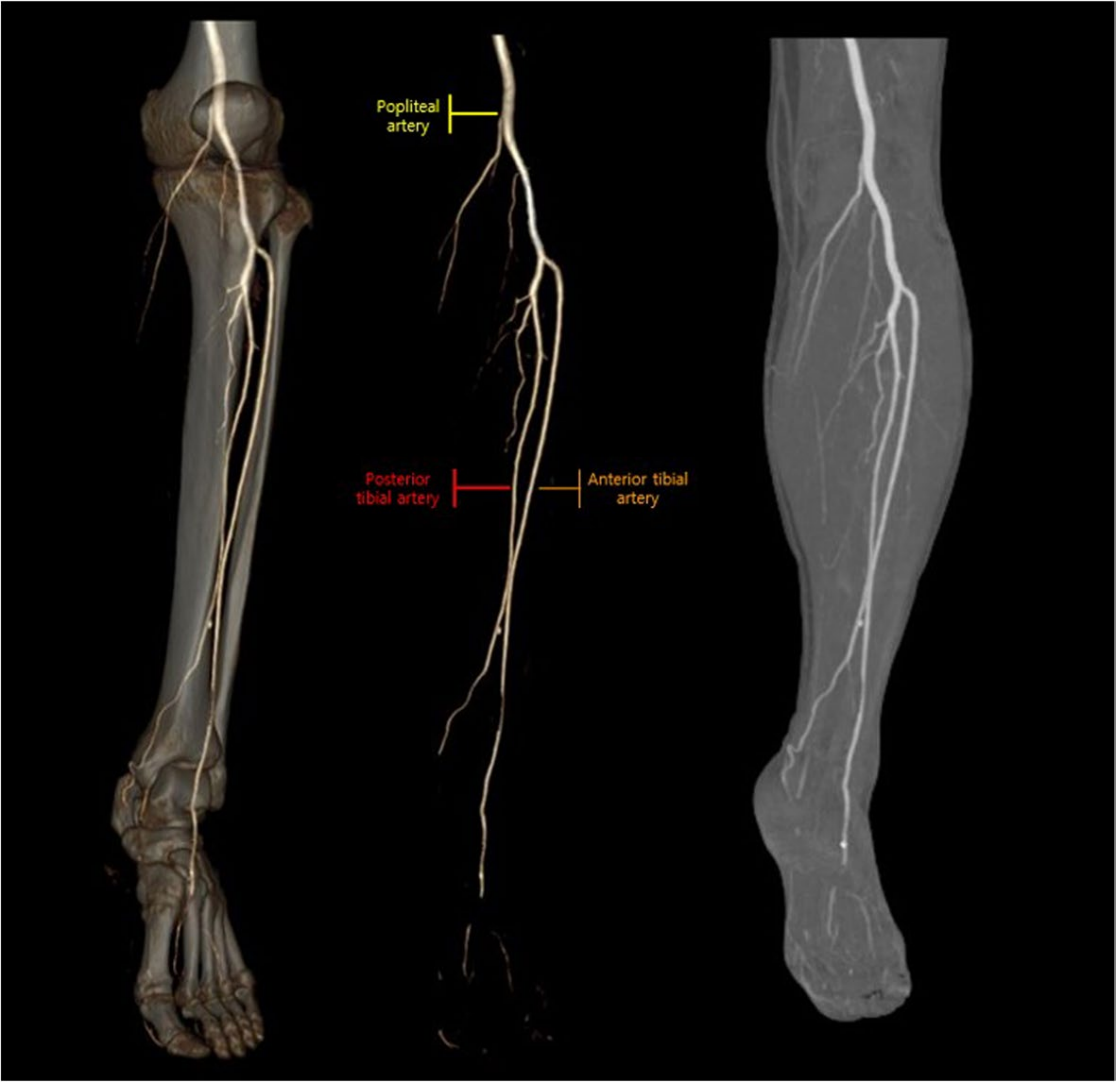
Angiography of lower extremities. No evidence stenosed in both lower extremity arteries

The surgical plan encompasses a conventional supra-omohyoid neck dissection (SOHND) in conjunction with a segmental mandibulectomy of the right mandible. This will be followed by a meticulous reconstruction utilizing a fibular free flap harvested from the left lower extremity. Preoperatively, angiography of both lower extremities revealed no noteworthy abnormalities, and the distribution of distal perforators in the fibula was duly confirmed through Doppler examination (Fig. 3).

**Fig. 4:**
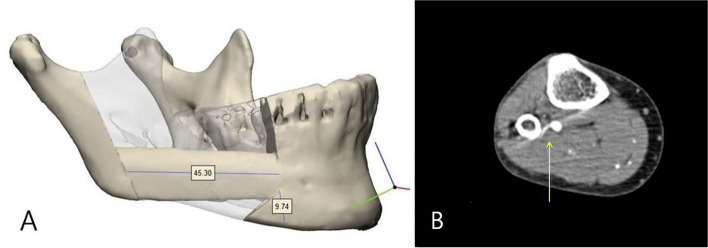
**A** Preoperative 3-dimentional reconstruction plan of the lesion. **B** Proximal perforator marked by yellow arrow

The reconstruction process was planned with the aid of a 3D surgical guide, facilitating the precise implementation of the fibular free flap technique (Fig. 4). Following the application of a tourniquet, an incision was made along the length of the left fibula, from top to bottom. The skin and subcutaneous tissue were then incised with careful attention to the perforator. After the incision, the underlying subcutaneous tissue was then delicately dissected. The soleus muscle was widely dissected, and the proximal perforator branch passing through the soleus muscle was preserved in the proximal part of the fibula. An attempt was made to assess the fibula’s shape and identify the distal perforator; however, no discernible perforator branch originating from the peroneal artery was observed in the distal portion. This led to the confirmation of a deficiency in the distal perforator, attributed to anomalous vascular anatomy. As an alternative approach, the decision was made to harvest a free fibular flap using the proximal perforator located on the upper part of the fibula. Subsequently, the surgical procedure was carried out accordingly.

**Fig. 5:**
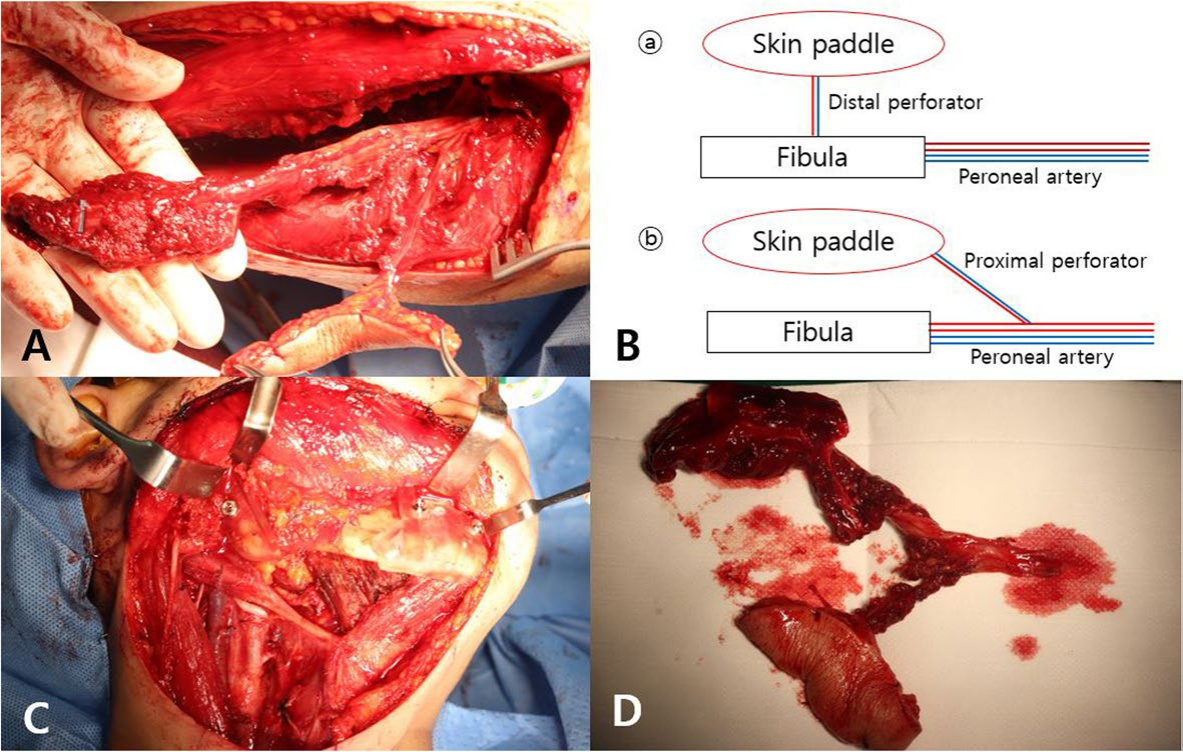
Procedure of fibular harvest. **A** Flap harvest with proximal perforator. **B** Diagram comparing anatomical variants (**ⓑ**) with typical case (**ⓐ**). **C** Proximal musculocutaneous perforator flap (**D**). Precisely reshaped fibular flap with 3-dimentional surgical guide model

First, the preserved proximal perforator was thoroughly reidentified and dissected. Subsequently, the fibula was resected using a bone cutter, resulting in the formation of a flap measuring 45.30 mm in size. The harvested flap was precisely reshaped into an appropriate angle and shape using a 3D guide model and then adjusted to fit the mandibular defect. Microvascular anastomosis of the flap and cervical vessels was performed using a microscope, and the suture of the flap was performed satisfactorily. After harvesting the flap, primary suture was performed on the donor site (Fig. 5).

**Fig. 6:**
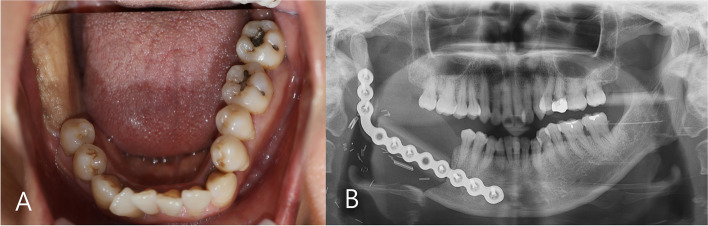
Postoperative 4-month follow-up. **A** Intraoral skin paddle maintained in good condition. **B** Panoramic radiography view indicated favorable healing. Fibular bone had satisfactorily adjusted with residual mandible and restored well

The patient was discharged after 18 days of postoperative hospital stay. During the 6-month follow-up visit, normal healing progression was noted without any abnormal findings. The intraoral flap maintained good condition, and no signs of internal metastasis or recurrence were observed. Computed tomography performed at 1 and 4 months postoperatively showed no evidence of recurrence of the lesion or lymph node metastasis (Fig. 6).

## Conclusions

In oral cancer surgery, various reconstructive approaches are used in surgically removed defect, depending on the extent and size of the soft and hard tissues involved. Flaps are categorized based on anatomical location and blood supply. The fibular flap was introduced by Taylor et al. in 1975 for tibial fractures [4] and reported its superiority [5]. Starting with Hidalgo’s report on the reconstruction of mandibular defects in 1989 [6, 7], it was used for defects in the oral and maxillofacial region. The fibular flap can be transplanted to a defect of up to 20 cm, providing a sufficient length of bone flap, and the blood supply of the flap is well maintained even when the size of the flap is large [8]. Additionally, the use of a tourniquet during dissection confers several advantages, including reduced blood loss and enhanced visibility within the surgical field, and allows direct suturing of the donor site even when the size of the flap is large.

In addition, the donor site complications are minimal and stable, and the large diameter of the peroneal artery makes anastomosis with cervical vessels relatively easy during microsurgery.

Additionally, since the donor site is located far away from the head and neck area, there is an advantage that two teams can operate simultaneously on both the donor and recipient sites. Due to these advantages, fibular flap is considered primarily during oral and maxillofacial reconstruction when reconstruction of a large defect in the mandible is required.

In 1987, Taylor anatomically described the perforator of the human body, and in 1989, Koshima introduced the perforator flap based on the musculocutaneous type. In the 2000s, with the development of microsurgical techniques, flaps based on perforator began to be introduced for the reconstruction of oral and maxillofacial defects. Reconstruction with a perforator flap requires microsurgical techniques and has the disadvantage of prolonging the surgery time due to microvascular anastomosis. However, perforator flaps have a lower thickness, allowing for more free tissue placement, and the perforator flaps are easier to handle, resulting in satisfactory results after reconstruction.

The average number of perforators originating from the peroneal artery is 4.8 [9], and most perforators range in size from 0.5 to 1 mm. In addition, since the majority of the perforators distributed in the fibula are located in the seventh or eighth position from the top when the tibia is divided, the primarily harvested site for fibular free flap during the harvest process is 8 to 12 cm above the ankle, which has a good blood supply [10].

The peroneal artery mainly supplies blood to the distal third of the fibula, and most perforators branch from the peroneal artery. However, it has been reported that the perforator of the fibula branches from sources other than the peroneal artery [11], rarely branching separately from the anterior tibial artery or popliteal artery [12–14]. There are also cases where the perforator is absent [15–17].

The skin paddle of fibula free flap receives blood supply from septocutaneous or musculocutaneous perforators originating from peroneal artery. The distal perforator is mostly a septocutaneous flap, whereas the proximal perforator is a musculocutaneous flap. Several cadaveric and radiographic studies concluded critical vascular anomalies in 10% of population, which can lead to failure of flap survival with ischemia on donor site [12].

For these reasons, it is essential to check the distribution of blood vessels in the lower extremities through angiography and Doppler ultrasonography before surgery [18]. Most (70–96%) of the perforators in the distal third of the fibula are septocutaneous type, branching from the peroneal artery and comes out along the intermuscular septum to supply blood on flap [19, 20]. On the other hand, perforators mainly distributed in the proximal third of the fibula are in the form of musculocutaneous type [21, 22], which penetrate the soleus muscle and travel to the skin to supply blood circulation [23–25].

The perforators branching from the peroneal artery are mainly concentrated in the distal third of the fibula with better blood supply. Fibula free flap harvest is based on the distal perforator in this reason [6]. In particular, an average of one to three perforators were observed in the distal third of the fibula. On the other hand, fibula flap with proximal perforators is not considered as the primarily method, due to disadvantages that come with its proximal location and low usefulness [26]. When forming a fibular flap, preoperative angiography of the lower extremities is performed to identify the branches of the peroneal artery [27], and the location or distribution of the subcutaneous perforators can be confirmed through Doppler examination. Therefore, preparing surgery with preoperative identification of the location of the perforator before the incision is critical [28, 29]. The distal perforator, which mainly distributes in the distal third of the fibular flap, is responsible for the blood supply to the flap and is known to be relatively stable and has little anatomical variation [30].

However, as in this case, there may be cases where the distal perforator is not observed due to aberrant anatomical defect, and such vascular abnormalities sometimes show contradictory results to the preoperative Doppler examination [12]. This is an error that may occur in Doppler examination when a perforator overlaps with other arteries in the surrounding area, making it difficult to rely solely on Doppler examination results. For this reason, clinicians should consider the possibility of identifying a defect in the perforator after incision of the skin, as in this case, and understand alternative approaches to flap formation [9].

In this case, by using a proximal flap design, the reconstruction was proceed as planned. The conventional fibula flap is connected with bone by the posterior intermuscular septum, and it is difficult to inset due to limitation of movement between the bone and skin paddle if defect is complex. This can also lead to failure to offer sufficient soft tissue volume. In contrast, the proximal perforator skin paddle is more flexible and provides extended soft tissue and offers advantages such as better visualization and possibility of chimeric flap elevation. The fibula free flap with proximal perforator is a reliable approach method in the reconstruction of extensive mandibular defects [8].

While reports exist regarding alterations in blood vessel dynamics during the formation of fibular free flaps and subsequent modifications in the harvesting process employing alternative vessels, documentation of perforator abnormalities remains limited [19, 25]. Nevertheless, it is imperative for clinicians to possess precise anatomical knowledge pertaining to blood vessel distribution in the microsurgical field and to be adept in responding to encounters with aberrant anatomical variations [26]. Though anatomic variations in perforator flaps are infrequent and distinctive, it is crucial to recognize the potential for deficiencies in the distal perforator. Moreover, preserving the proximal perforator identified during skin incision and comprehending flap formation with the proximal perforator, when necessary, are deemed pivotal factors in ensuring successful flap formation and a favorable prognosis in the presence of vascular abnormalities during the flap harvest process [31–33].

## Data Availability

The datasets used during the current study are available from the corresponding author on reasonable request.

## Abbreviations

SCC: Squamous cell carcinoma
MRI: Magnetic resonance imaging
FDG: Fluorodeoxyglucose
